# Ethanol toxicity differs depending on the time of day

**DOI:** 10.1371/journal.pone.0190406

**Published:** 2018-01-02

**Authors:** Luisa M. Vera, Carolina Bello, Juan F. Paredes, Greta Carmona-Antoñanzas, Francisco J. Sánchez-Vázquez

**Affiliations:** 1 Department of Physiology, Faculty of Biology, Regional Campus of International Excellence “Campus Mare Nostrum”, University of Murcia, Murcia, Spain; 2 Institute of Aquaculture, Faculty of Natural Sciences, University of Stirling, Stirling, United Kingdom; Karlsruhe Institute of Technology, GERMANY

## Abstract

Ethanol is one of the most commonly abused drugs and consequently its toxic and psychoactive effect has been widely investigated, although little is known about the time-dependent effects of this drug. In the present research zebrafish was used to assess daily rhythms in ethanol toxicity and behavioural effects, as well as the temporal pattern of expression of key genes involved in ethanol detoxification in the liver (*adh8a*, *adh5*, *aldh2*.*1* and *aldh2*.*2*). Our results showed marked differences in the mortality rate of zebrafish larvae depending on the time of day of the exposure to 5% ethanol for 1h (82% and 6% mortality in the morning and at night, respectively). A significant daily rhythm was detected with the acrophase located at “zeitgeber” time (ZT) = 04:22 h. Behavioural tests exposing zebrafish to 1% ethanol provoked a major decrease in swimming activity (68–84.2% reduction) at ZT2, ZT6 and ZT10. In contrast, exposure at ZT18 stimulated swimming activity (27% increase). During the day fish moved towards the bottom of the tank during ethanol exposure, whereas at night zebrafish increased their activity levels right after the exposure to ethanol. Genes involved in ethanol detoxification failed to show significant daily rhythms in LD, although all of them exhibited circadian regulation in constant darkness (DD) with acrophases in phase and located at the end of the subjective night. Taken altogether, this research revealed the importance of considering the time of day when designing and carrying out toxicological and behavioural tests to investigate the effects of ethanol, as the adverse effects of this drug were more marked when fish were exposed in the morning than at night.

## Introduction

Chronotoxicology is a discipline that studies the temporal variations in the presence and severity of adverse effects of drugs and other chemicals when administered to an organism at different times of the day [1]. The rhythmic physiology of organisms regulates the effectiveness of drug absorption, distribution, metabolism and excretion, which in turn determines its concentration in the blood and bioavailability, controlling the levels of cellular exposure to drugs and toxicants [2,3]. In fact, the assessments conducted in experimental animals under constant lighting conditions have proofed that many of these rhythmic detoxification mechanisms persist, indicating its endogenous nature and discarding potential direct effects of light on metabolic rhythms [4].

Ethanol (alcohol or ethyl alcohol) is one of the most widely abused drugs consumed in the world [5] and therefore its toxic and psychoactive effect has been thoroughly studied. Zebrafish (*Danio rerio*) has been proposed as an ideal vertebrate model to investigate the neurobehavioural effects of ethanol addiction [6] as well as the mechanisms underlying those effects [7–9] mostly because ethanol metabolism in zebrafish liver is similar to humans [10]. In addition, zebrafish can be easily kept and bred in the laboratory, providing readily access at the embryonic and larval stages for genetic and screening tests [11]. Besides, ethanol is a water-soluble substance that can be directly added to the fish tank, so zebrafish take up this hydrophilic compound constantly through the skin and gills, and consequently blood and brain ethanol levels remain stable [8]. Similarly to humans, current evidence suggests that ethanol metabolism in zebrafish liver comprises two detoxification steps by alcohol dehydrogenase (ADH) and aldehyde dehydrogenase (ALDH) [10]. Along this route, ethanol is converted first into acetaldehyde and then into acetic acid, the accumulation of these products being responsible for the negative physiological and behavioural effects of ethanol [12]. Reimers et al. [13] studied alcohol dehydrogenases in zebrafish, concluding that ADH8A is a functional class I alcohol dehydrogenase with high affinity for ethanol. However, despite ADH8B was also classified as a class I alcohol dehydrogenase based on phylogenetic analysis it does not metabolise ethanol. Moreover, class III ADH5 can also metabolise ethanol but its affinity for this substrate is low and it preferentially metabolises longer aliphatic and aromatic alcohols. Regarding aldehyde dehydrogenases, ALDH2 has a high affinity for acetaldehyde, the toxic oxidised product of alcohol, and it is primarily responsible for the conversion of this compound into non-toxic acetic acid [14].

Despite the relatively large number of studies focusing on the effects of ethanol [6,8,9,15], a limited amount of research has been devoted to investigate the dosing-time effects of this drug. Early studies in mice reported that ethanol was more lethal during the night than during daytime [16]. Longer episodes of loss of righting reflex were also detected when ethanol was administered at the beginning of the animals’ active phase (night) in comparison with the beginning of their resting phase (day), which was not correlated to differences in the activity of several cytochrome P450 enzymes [17]. In humans, Wilson et al. [18] first reported that morning consumption of alcohol resulted in higher peak blood concentrations, so at the time of rising from sleep alcohol reached the highest peak plasma values and the shortest “time-to-peak” [19,20].

Fish in general and zebrafish in particular have become widely recognised model organisms in chronobiology studies [21]. In fact, the use of zebrafish in chronobiological and biomedical research offers an advantage over nocturnal rodents, making zebrafish a useful model organism when extrapolating results to humans. Therefore, in the present study zebrafish was used to assess daily rhythms in ethanol toxicity and psychoactive (behavioural) effects, as well as the light- and clock-controlled expression patterns of genes involved in ethanol and acetaldehyde metabolism in liver (*adh8a*, *adh5*, *aldh2*.*1* and *aldh2*.*2*).

## Material and methods

### Animals & housing

The toxicity and behavioural trials were carried out in the Chronobiology Laboratory at the Faculty of Biology of the University of Murcia. Zebrafish larvae (5 days post-fertilisation, dpf) were used to investigate chronotoxicity of ethanol. Fertilised eggs from wild-type stock zebrafish (short-fin phenotype) were collected within 2 h after laying, and aliquots of 30 eggs were transferred into sterile Petri dishes (85x10mm) filled with embryo medium according to standard methods [22]. Pairs of Petri dishes (N = 60) were incubated for 5 days in a rack of six 9 L thermostat-controlled aquaria kept at 28°C.

For behavioural trials, a total of 84 mixed sex wild-type AB adult zebrafish (0.40 ± 0.10 g body weight) were obtained from a local provider (Jumipez SL, Murcia, Spain). During a two-week acclimation period, zebrafish were housed in glass aquariums (60cm x 40cm x 30cm) equipped with filter pumps and placed in a chamber where the temperature was kept constant at 26.0 ± 0.5°C and photoperiod set at 12 h: 12 h light-dark (LD). Light was provided by fluorescent tubes (F15W/GRO, Sylvania Gro-Lux, Germany), being the intensity at the water surface 700 lux. Fish were fed once a day *ad libitum* a commercial diet (Nutron Hi-Fi; Prodac, Italy) at random times during daytime.

For gene expression analysis, 84 mixed sex wild-type AB adult zebrafish (0.42 ± 0.13 g body weight) were obtained from the University College of London Fish Facility (London, UK) and housed in an isolated fish laboratory at the Institute of Aquaculture of the University of Stirling (Stirling, Scotland). Experimental fish were randomly allocated to twelve 11 L plastic tanks (35.6 x 23.4 x 22.8 cm) (Geo Extra Large Tank, Ferplast, Italy) (n = 7 fish/tank), each one equipped with an individual filter (PF Mini Internal Power Filter, Interpet, UK) and supplied with filtered and dechlorinated tap water. During the acclimation period, the photoperiod was set at 12 h: 12 h light-dark (LD) and temperature was kept constant at 25°C throughout the trial using water heaters (H2 Therm 15W Micro Aquarium Heater, Tropical Marine Centre, UK). Fish were hand-fed once a day *ad libitum* a commercial diet (Otohime B2 360–650 μM, Marubeni Nisshin Feed Co., Ltd., Japan) at random times during daytime over a two-week acclimation period and during the trial. The walls of all aquaria were covered with black plastic sheets to prevent animals from seeing each other.

### Experimental design

The procedures in experiments 1 and 2 complied with the Guidelines of the European Union (2010/63/UE) and the Spanish legislation (RD 53/2013 and law 32/2007) under the approval of the Animal Health Service of the Region of Murcia (Permit Number: A13150103) and under the supervision of Prof. F.J. Sánchez-Vázquez, holding a Category C license for animal research (responsible for directing animals experiments), according to the Spanish legislation (RD 53/2013). Experiment 3 complied with the Guidelines of the European Union (2010/63/UE) and the Animal (Scientific Procedures) Act 1986 UK and was approved by the Animal Welfare and Ethical Review Body (AWERB) of the University of Stirling for the use of animals in research.

#### Experiment 1: Daily rhythm of ethanol toxicity in zebrafish larvae

Zebrafish a total of 420 larvae aged 5 days post-fertilisation (dpf) were exposed to 5% ethanol in fresh embryo medium (v/v) for 1 h every 4 h during a 28 h period, at “Zeitgeber Times”(ZT) 2, ZT6, ZT10, ZT14, ZT18, ZT22 and ZT2b (ZT2 of the following day) (lights onset at ZT0). This concentration and exposure time was decided based on previous screening tests carried out in our laboratory, showing very low mortality rates for exposures to 4% ethanol at ZT2-ZT18 (1–8%) and high variability between replicates (S1 File). At each sampling point 6 independent Petri dishes containing 10 larvae were used to carry out ethanol exposure. A control group exposed to fresh embryo medium was also included at each sampling point. Petri dishes were kept in a 60 L thermostat-controlled aquarium at 28°C. During the exposures larvae were monitored to check for immobility, absence of heartbeat and lack of reaction to mechanical stimulus, according to the OECD guidelines for testing of chemicals in fish larvae (OECD TG 210). To this end the presence or absence of heartbeat was determined with a microscope (Leica EZ4D, Germany). Zebrafish larvae are transparent and therefore their heart rate can be easily observed and measured. A total of 175 larvae were found dead during the experiment. Dead larvae were removed as soon as observed, according to OECD Test Guideline 212. At the end of each exposure, total larval mortality rate was assessed and all surviving larvae were immediately euthanized by anaesthetic overdose and death confirmed under the microscope.

#### Experiment 2: Effect of sublethal ethanol concentrations on locomotor activity of adult zebrafish

To investigate the existence of daily rhythmicity in the effect of ethanol on zebrafish activity, a total of 84 adult fish were exposed to 1% ethanol in dechlorinated water (v/v) for 15 min every 4 h during a 24 h cycle, at ZT2, ZT6, ZT10, ZT14, ZT18 and ZT22 (n = 14 fish/ZT). Both ethanol concentration and duration of exposure were chosen based on previous tests and published data [11]. Water temperature was kept constant throughout the trials at 26°C. To evaluate the effect of ethanol on locomotor activity, fish were filmed prior to, during and after exposure. For this, at each ZT two 8 L tanks (n = 7 fish/tank) were divided into 7 individual compartments with methacrylate separators that were pierced to allow water circulation. The experimental conditions were the same and the experiment was run in both aquaria at the same time. Zebrafish were netted from their group housing tanks and placed in their test tanks, as described previously [23]. Then activity was recorded during the 15 min before exposure (pre-exposure activity), 15 min during ethanol exposure and 25 min after exposure to record the recovery phase, for which clean water was provided. Fish were fasted for 24 h prior to the experiment and access to the experimental laboratory was restricted during the course of the trial to avoid fish disturbance. Fish were filmed with webcams (Webcam C250, M/N: V-U0003, Logitech, Switzerland) which were adapted for infrared recording at night by replacing the UV filter located in front of the lens with a negative film. During the day, light was provided with a fluorescent bulb (F15W/GRO, Sylvania Gro-Lux, Germany), whereas at night infrared LED lamps were used (LEDs monocolor λ = 950nm, mod. L-53F3BT, 5 mm). These lamps were not perceived by fish but allowed video-filming.

#### Experiment 3: Gene expression of ethanol metabolising enzymes

To investigate daily rhythms of gene expression in LD, 42 adult fish were housed in six 11 L tanks (n = 7/tank) were acclimatised for 2 weeks, fasted for one day and then sacrificed by lethal anaesthesia (MS-222, 1000 ppm, PHARMAQ, UK) every 4 h during a 24 h period, at “Zeitgeber Times” (ZT) 2, ZT6, ZT10, ZT14, ZT18 and 22 (1 tank/ZT). Liver samples were obtained from each fish and preserved in RNALater (Sigma-Aldrich, Poole, UK). In darkness conditions, sampling was performed using dim red light attached to the dissecting microscope.

To determine the existence of circadian rhythms of gene expression, the remaining 42 experimental fish (n = 7/tank, 6 tanks) were kept under an LD cycle for an additional week and then lights were switched off at ZT0. Fish were fasted and kept in continuous darkness (DD) for 24 h and then sampled, starting at circadian time (CT) 2 (onset of the subjective day). Samples were obtained every 4 h during a 24 h cycle (at CT2, CT6, CT10, CT14, CT18 and CT22). From each fish, liver samples were also collected in RNALater.

Liver samples were homogenised in 1 mL of TRIzol (Invitrogen, UK) and total RNA extracted in accordance with the manufacturer’s instructions. RNA pellets were rehydrated in DNase RNase-free distilled water (Merck Millipore) and total RNA concentration determined using an ND-1000 Nanodrop spectrophotometer (Labtech Int., East Sussex, UK). RNA integrity was assessed by agarose gel electrophoresis.

The relative expression of 4 genes involved in ethanol metabolism was determined in liver from fish of all treatments: *aldehyde dehydrogenase 2*.*1* (*aldh2*.*1*), *aldehyde dehydrogenase 2*.*2* (*aldh2*.*2*), *alcohol dehydrogenase 5* (*adh5*) and *alcohol dehydrogenase 8a* (*adh8a*) (Table 1). In addition, the gene expression of references genes (*bactin1*, *slc25a5*, *b2m*, *elf1a*, *rpl13*) was determined to normalise target gene expression values. The software PRIMER3 [24] was used to design new sets of primers and their target specificity was checked *in silico* using Blast (NCBI) (Table 1). cDNA was reverse transcribed from 1 μg of total RNA using QuantiTect Reverse Transcription kit (Qiagen Ltd., Manchester, UK). The resulting cDNA was diluted 20-fold with Milli Q water and 2.5 μL of each sample was used in combination with 300 nM of each primer and 5 μL of Luminaris Color HiGreen qPCR Master mix (Thermo Fisher Scientific, MA, USA) to reach a final PCR volume of 10 μL. Reactions were run in a Mastercycler RealPlex 2 thermocycler (Eppendorf, UK) programmed to perform the following protocol: UDG pre-treatment at 50°C for 2 min preceded thermal cycling, which was initiated at 95°C for 10 min, followed by 40 cycles with a denaturing step at 95°C for 15 s, annealing for 30 s at Ta according to Table 1 and extension at 72°C for 15 s. The amplification cycle was followed by a temperature ramp with 0.5°C increments ranging between 60°C and 90°C for melt-curve analysis to verify that no primer-dimer artefacts were present and only one product was generated from each qPCR assay. Amplifications were carried out including systematic negative controls containing no cDNA (NTC, no template control) and omitting reverse transcriptase enzyme (-RT) to check for DNA contamination. In addition, the qPCR product sizes were checked by agarose gel electrophoresis and the identity of random samples was confirmed by sequencing (GATC Biotech, Germany). No primer-dimer occurred in the NTC. Gene expression quantification was achieved by including a parallel set of reactions containing serial dilutions from all pooled cDNA experimental samples and assigning each dilution the appropriate value of relative units (RUs) of target genes by a normalization factor obtained from the expression of the most stable reference genes. As a result, an estimated number of relative copies, corrected for the efficiency of the reaction, was automatically calculated for each sample.

**Table 1:** Primers used for qRT-PCR.

Gene	Forward sequence (5’-3’)	Reverse sequence (5’-3’)	Amplicon	Ta	Accession number	Reference
***aldh2*.*1***	GAGTTGGGCGAGTATGGACT	TTAACGTGGCAATTCGTGACT	126 bp	60°C	NM_200490.1	New design
***aldh2*.*2***	TGAGATGGGCGAGTATGGAC	TGATCATTCGGCTGCTTCTT	122 bp	60°C	NM_213301.2	New design
***adh5***	CCCGGACAAGTTTGAAATCG	TCCACCAGCACCTCCTGAAT	93 bp	60°C	NM_131849.2	New design
***adh8a***	GAAGGCCAAGGTGTTTGGAG	CCGTGCACTCGATTGAGAAG	122 bp	60°C	NM_001001946.4	New design
***bactin1***	CGAGCAGGAGATGGGAACC	CAACGGAAACGCTCATTGC	102 bp	56°C	AF057040	[25]
***slc25a5***	AAGCGACACCTCTCCAAGAA	TAGCATGTTGCACCTGAAGC	153 bp	56°C	NM_173247	New design
***b2m***	AGGATTGTCTGCTTGGCTCTCT	GGAGTGGAGACTTTCCCCTGTAC	110 bp	56°C	NM_131163	[26]
***elf1a***	CCTCTTGGTCGCTTTGCTGT	CTTGGTCTTGGCAGCCTTCT	129 bp	57°C	AY422992.1	New design
***rpl13***	TCTGGAGGACTGTAAGAGGTATGC	AGACGCACAATCTTGAGAGCAG	148 bp	56°C	NM_212784	[26]

### Data analysis

Daily rhythms in larvae mortality and gene expression were analyzed using Cosinor analysis, which was performed using “*Ritme*" software (Prof. A. Díez-Noguera, University of Barcelona, Spain) to determine whether the daily expression of the studied genes fitted the cosine function: *Y* = *M* + *A* * [Cos (Ω*t* + Φ)], where *M* is mesor, *A* is amplitude, Ω is angular frequency (360°/24h for the circadian rhythms) and Φ is acrophase. Statistical differences in mortality and gene expression between different sampling times were analysed by one-way ANOVA (ANOVA I), followed by Tukey’s post hoc test. Prior to ANOVA I, mortality percentages were transformed using the square root arcsine transformation [27].

The video-recordings were analysed using the specialised software Fish Tracker [28] to measure locomotor activity levels and determine fish position in the water column. This software tracks each fish position every second during the experiment and generates a file that can be exported to Microsoft Excel for further analysis. To assess how activity changed at each ZT, the mean activity was calculated for each phase of the trial (pre-exposure, ethanol exposure and recovery) and the existence of statistical differences between these means was checked by a GLM repeated measures, in which the within-subjects factor was “time”, with 3 levels. Then, a Least Significant Difference (LSD) test was used to perform multiple pairwise comparisons between the mean activity levels in each phase. Statistical differences in activity levels during ethanol exposure between different ZTs were analyzed by one-way ANOVA (ANOVA I) followed by Tukey’s HSD post-hoc test. Statistical analyses were performed using SPSS v.19 software (IBM, Armonk, NY), with significance level fixed at *p* < 0.05.

## Results

### Daily rhythm of ethanol toxicity in zebrafish larvae

**Fig 1:**
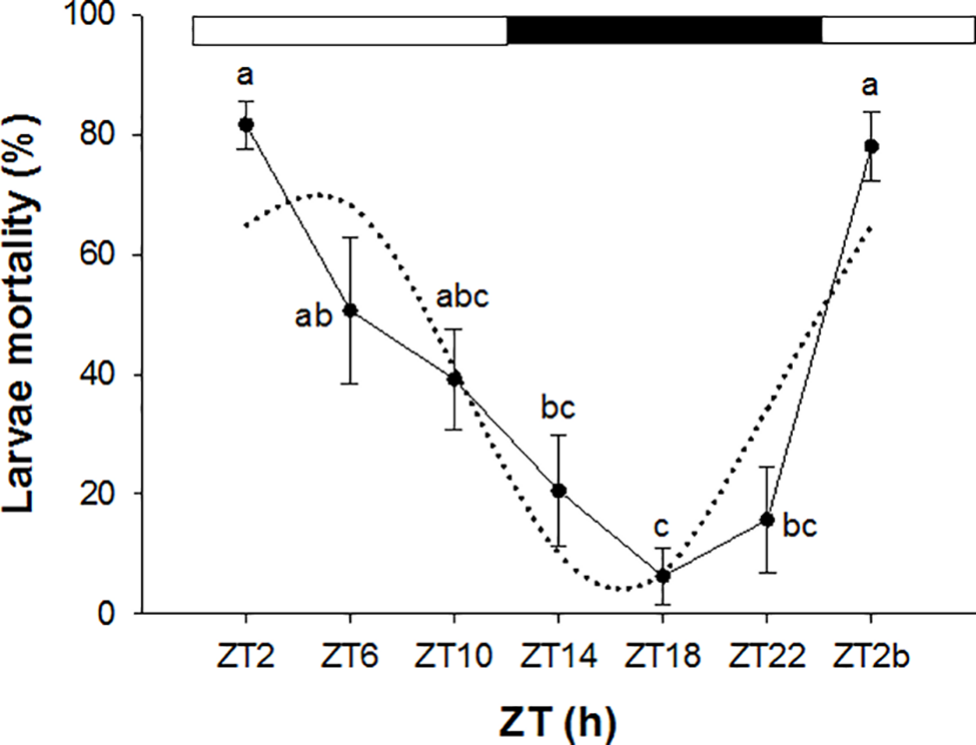
Daily rhythm of mortality in zebrafish larvae exposed to 5% ethanol for 1 h. White and black bars at the top of the graph indicate the light and dark phases, respectively. Data are shown as the mean ± SE (n = 60 larvae/ZT). Superscript letters indicate statistically significant differences (ANOVA I, p<0.05). The dotted black line represents the sinusoidal function determined by Cosinor analysis. ZT, zeitgeber time.

The mortality rate of zebrafish larvae exposed to 5% ethanol showed striking differences between sampling times (ANOVA I, *p* < 0.05): the highest mortality rate was observed at the beginning of the photophase on both Day 1 (ZT2) and 2 (ZT2b) (81.7% and 78.0%, respectively), thereafter mortality rate gradually decreased along the day and reached the lowest rate (6.3%) in the middle of the dark phase (ZT18) (Fig 1). A significant daily rhythm was also detected with the acrophase located at ZT = 04:22 h (cosinor, *p* < 0.05). No mortality was recorded in control larvae at any ZT.

### Effect of sublethal ethanol concentrations on locomotor activity of adult zebrafish

**Fig 2:**
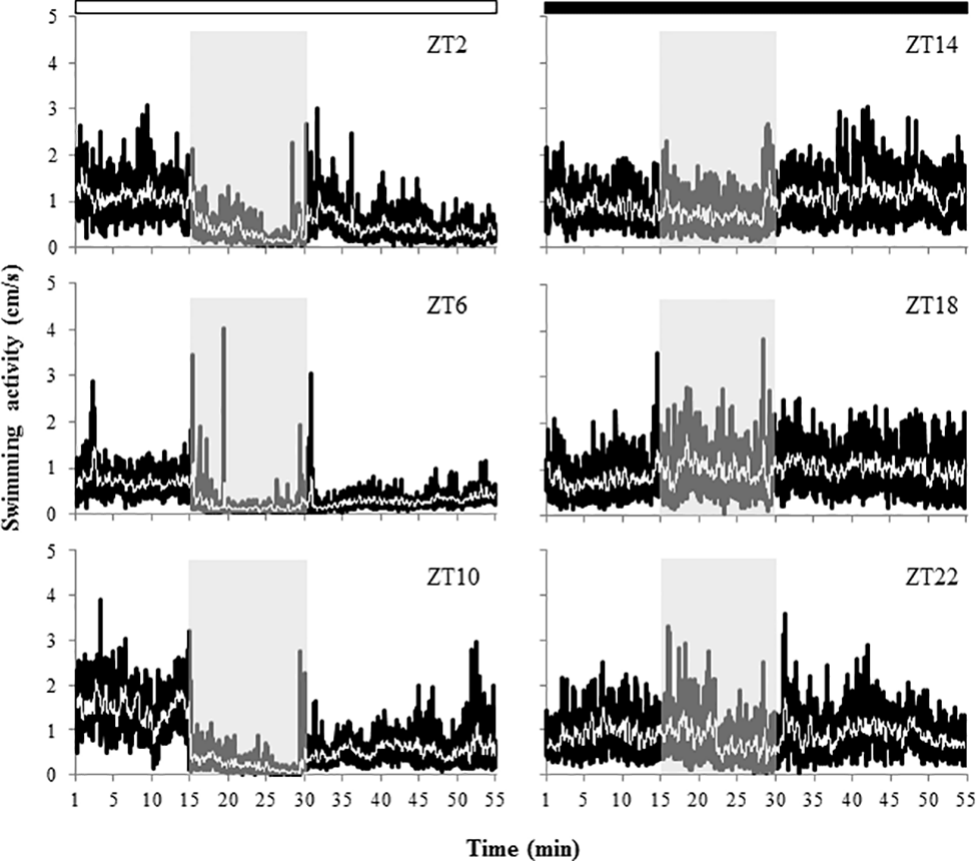
Average locomotor activity (cm/s) of adult zebrafish before, during and after exposure to 1% ethanol for 15 min at different times of the day (tests at ZT2, ZT6, ZT10, ZT14, ZT18 and ZT22) (n = 14 fish/ZT). Grey boxes indicate the exposures to ethanol. Black lines indicate the fish activity every 1 s, whereas the white line represents the mobile average of the original data every 60 s. White and black bars at the top of the graph indicate the light and dark phases, respectively. ZT, zeitgeber time.

The effect of 15 min exposure to 1% ethanol on locomotor activity showed also daily variations. In particular, the effects were more severe during the photophase than during the dark phase. At ZT2, ZT10, ZT14 and ZT22 fish reduced significantly their activity during ethanol exposure (GLM repeated measures, *p* < 0.05) (Fig 2). However, the overall reduction of activity during ethanol exposure was more marked at ZT2, ZT6 and ZT10 (-68%, -73% and -84.2%, respectively) than at ZT14 and ZT22 (-18% and -14%, respectively) (Table 2). Conversely, GLM repeated measures followed by LSD test showed that ethanol increased locomotor activity of zebrafish at ZT18 (+27%) and that this increase continued during the recovery phase (*p* < 0.05) (Fig 2) (Table 2). During the recovery phase, when clean water was provided, the activity levels of zebrafish exposed during the photophase increased but remained lower than the pre-exposure ones. However, at ZT14 and ZT18, activity levels during the recovery phase were higher than those observed prior to ethanol exposure, whereas at ZT22 no significant differences were found in activity levels between the pre-exposure and the recovery phases (GLM repeated measures followed by LSD test, *p* < 0.05) (Table 2) (Fig 2). Regarding differences in activity levels during exposure between ZTs, the lowest levels were observed when fish were exposed to ethanol at ZT6 and ZT10, followed by ZT2. However, the highest activity levels were registered when the exposure was carried out at ZT18 (middle of the dark phase) (ANOVA I, Tukey HSD test, *p <* 0.05).

**Table 2:** Activity levels (cm/s) in zebrafish before (pre-exposure), during and after exposure (recovery) at different times of the day.

	ZT2	ZT6	ZT10	ZT14	ZT18	ZT22
Pre-exposure	1.11±0.01^c^	0.72±0.01^c^	1.46±0.02^c^	0.90±0.01^b^	0.78±0.01^a^	0.91±0.01^b^
Exposure	0.36±0.01^aB^	0.19±0.01^aA^	0.23±0.01^aA^	0.74±0.01^aC^	0.99±0.02^bD^	0.78±0.02^aC^
Recovery	0.54±0.01^b^	0.26±0.01^b^	0.54±0.01^b^	1.15±0.02^c^	1.10±0.01^c^	0.95±0.02^b^

All values are expressed as the mean ± standard error (SE). Superscript lowercase letters indicate statistically significant differences in activity levels between phases during the behavioural tests carried out at each ZT (GLM repeated measures, LSD test *p*<0.05). Superscript capital letters imply statistical differences in activity levels during ethanol exposure between different ZTs (ANOVA I, Tukey HSD test, *p*<0.05).

**Fig 3:**
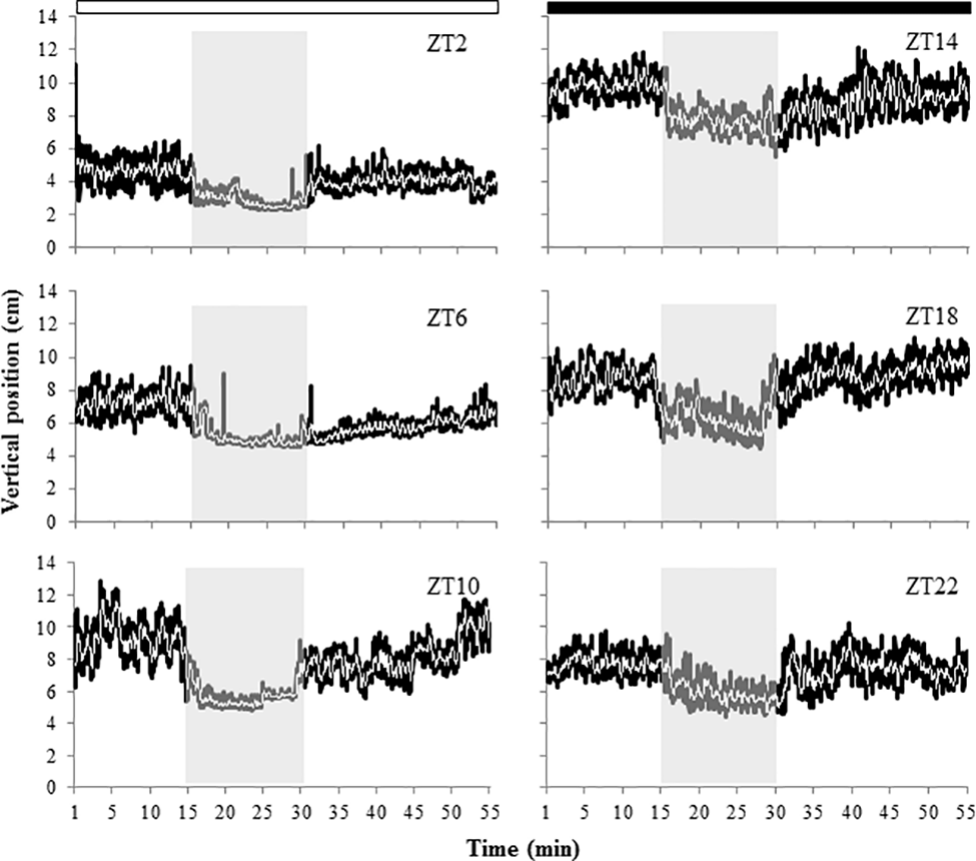
Average position (cm) of adult zebrafish in the water column before, during and after exposure to 1% ethanol for 15 min at different times of the day (tests at ZT2, ZT6, ZT10, ZT14, ZT18 and ZT22) (n = 14 fish/ZT). Graph definitions as given for Fig 2.

Ethanol exposure also had an effect on the fish vertical position in the water column. Thus, at ZT2, ZT6 and ZT10, zebrafish swam between 6–10 cm from the bottom of the aquarium prior to ethanol exposure. However, when ethanol was added to the tanks fish movements up and down in the water column were dramatically reduced and fish remained closer to the bottom. After the exposure, fish resumed their vertical movements at ZT2 and ZT10, whereas at ZT6 fish remained lower in the water column for the whole recovery phase. At ZT14, ZT18 and ZT22 (night hours) fish position during ethanol exposure was also lower in the water column but vertical movements were not suppressed, and once clean water was provided, fish quickly resumed their swimming activity closer to the water surface (Fig 3).

### Gene expression of enzymes involved in ethanol detoxification

**Fig 4:**
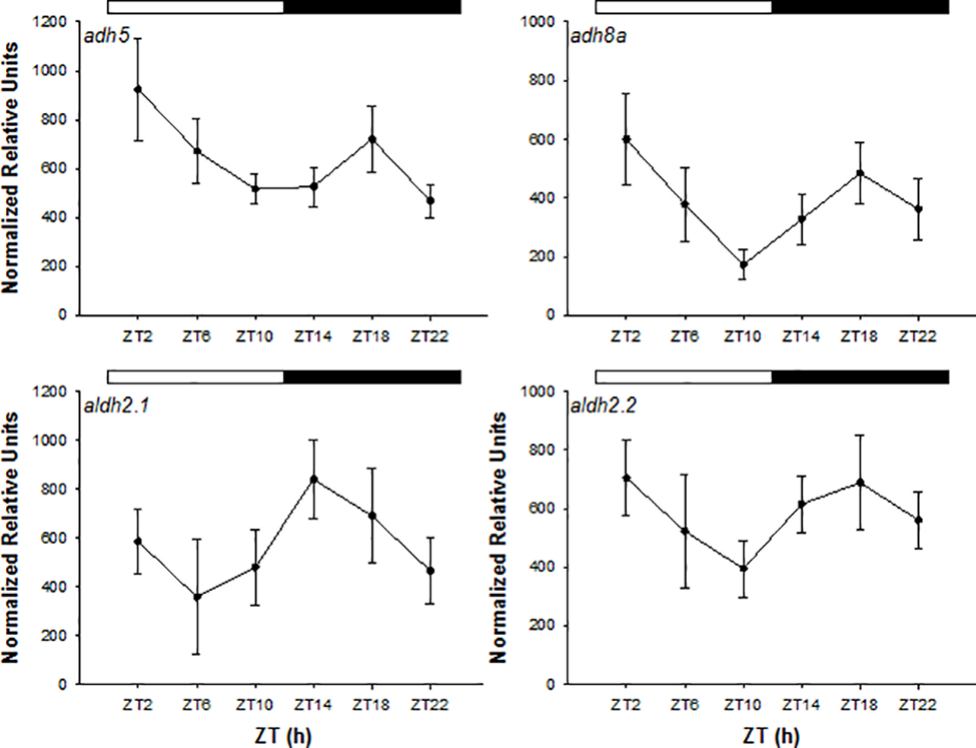
Relative expression of ethanol metabolising genes in the liver of zebrafish kept in LD. The white and black bars at the top of the graph indicate the photophase and darkness periods, respectively. Data are shown as the mean relative units (RU) ± SE (n = 7). ZT, zeitgeber time.

**Fig 5:**
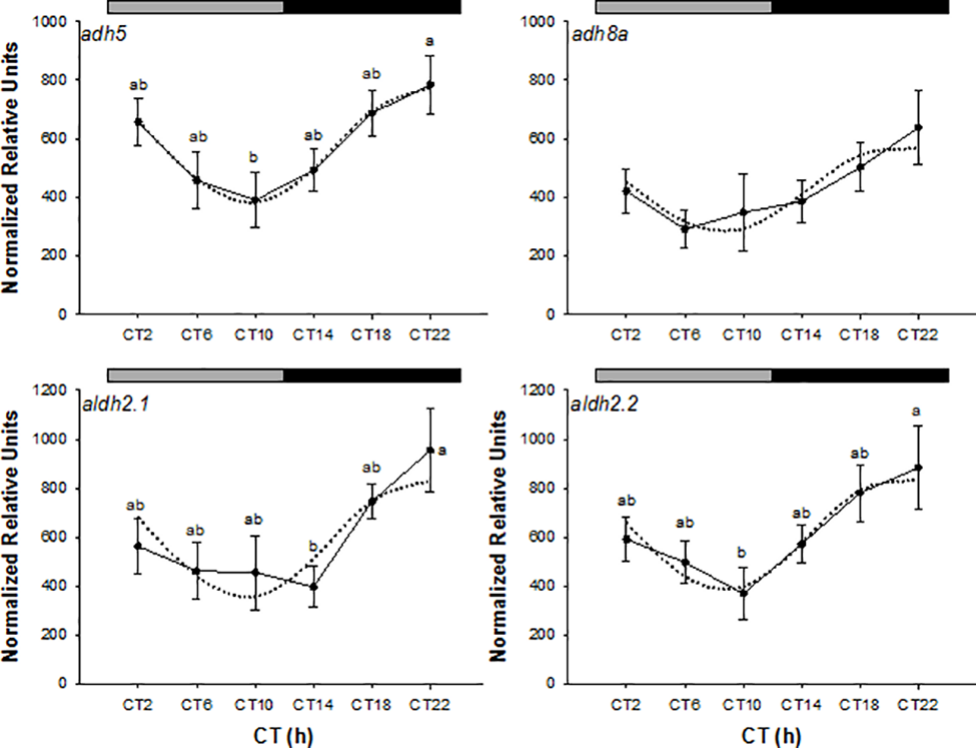
Relative expression of ethanol metabolising genes in the liver of zebrafish kept in DD. The grey and black bars at the top of the graph indicate the subjective photophase and darkness phase, respectively. Data are shown as the mean relative units (RU) ± SE. (n = 7). Superscript letters indicate statistically significant differences (ANOVA I, p<0.05). The dotted black line represents the sinusoidal function determined by Cosinor analysis. CT, circadian time.

Under LD, the expression of all genes investigated showed variations between sampling points though differences were not statistically significant (ANOVA, *p>0*,*05*) (Fig 4). However, cosinor analysis in DD revealed that the expression of all genes (*adh5*, *adh8a*, *aldh2*.*1* and *aldh2*.*2*) showed circadian rhythmicity (*p* < 0.05), with their acrophases in phase and located between CT = 20:36 h and CT = 21:38 h, at the end of the subjective night (Table 3). In addition, the expression of *adh5*, *aldh2*.*1* and *aldh2*.*2* also displayed significant statistical differences between time points, peaking at CT22 in all cases and showing the lowest levels in the CT10-CT14 (ANOVA I, *p* < 0.05) (Fig 5).

**Table 3:** Parameters of the cosine function calculated by Cosinor analysis (p<0.05) for diel expression of ethanol detoxification genes in zebrafish exposed to a light-dark (LD) cycle or continuous darkness (DD).

Gene	Light regime	p value	Mesor (RU)	Amplitude (RU)	Acrophase (ZT/CT hours)
***adh5***	LD	NS			
	DD	<0.01	577.9±35.0	198.0±62.0	21:38±01:19
***adh8a***	LD	NS			
	DD	<0.05	429.7±40.4	146.4±72.2	20:36±02:46
***aldh2*.*1***	LD	NS			
	DD	<0.01	593.7±52.2	240.3±94.0	21:22±01:41
***aldh2*.*2***	LD	NS			
	DD	<0.01	615.4±45.2	230.9±81.1	20:42±01:30

All parameters are expressed as the mean ± standard error (SE).

## Discussion

Zebrafish has become an established model to investigate ethanol effects at different physiological levels, including development, behaviour and toxicological response, showing that this drug causes both neurobehavioural and teratogenic effects [8,29,30]. However, this research provides novel evidence about the time-dependent effects of ethanol in this species. Our data evidences a daily rhythm of ethanol at different levels: in acute toxicity (lethality) and in behavioural responses. In addition, circadian rhythmicity in the expression of key genes involved in ethanol detoxification was also found, providing a metabolic background to understand the rhythmic effects of ethanol.

In zebrafish larvae, our results showed that ethanol exposure resulted in striking differences in the mortality rate depending on the time of the day in which the administration of the drug was carried out. Exposure to 5% ethanol for 1 h caused around 80% mortality of larvae in the morning (ZT2 and ZT2b), whereas at ZT18 (middle of the night) mortality rate dropped to 6%. The comparable mortality rate of larvae at ZT2 (5dpf) and ZT2b (6 dpf) showed that the time-dependent differences observed in our study were due to a daily rhythm rather than to developmental effects. Similar time-dependent effects were previously observed in toxicity tests carried out with anaesthetics (MS-222 and eugenol) in adult zebrafish. For both compounds higher mortality rates were reported when fish were exposed during the day than at night [23]. Likewise, in gilthead sea bream (*Sparus aurata*), a marine fish species, MS-222 also showed higher toxicity during daytime [28]. Results from our behavioural test using zebrafish adults were also in accordance with the daily rhythm in ethanol lethality observed in larvae. Thus, the effect of 1% ethanol was more severe during the photophase (zebrafish active phase) than during the dark phase (resting phase). In particular, fish showed a significant reduction in swimming activity when ethanol exposure was carried out at ZT2, ZT6, ZT10, ZT14 and ZT22, although this reduction was less marked at ZT14 and ZT22 (beginning and end of the darkness phase). In addition, fish moved towards the bottom of the tank during ethanol exposure and, during the photophase, the up and down movements in the water column were inhibited, which is in agreement with studies reporting that 1% ethanol reduced turning behaviour and vertical movements in zebrafish [9] and caused bottom dwelling [31]. However, when fish were exposed to the same concentration of ethanol at ZT18, an increase of activity levels was observed. In fact, significant lower levels of activity were found when zebrafish were exposed to ethanol during the photophase than during the dark phase. Previous research has showed that acute exposure to low and moderate concentrations of ethanol (e.g. 0.25 and 0.50% v/v) increases locomotor activity and reduces anxiety-like behaviour in zebrafish whereas higher concentrations (e.g. 1% v/v) inhibit locomotor activity and increase anxiety-like behavioural response [11], due to the sedative effects of ethanol which can cause general slowing and impaired coordination and swimming [32]. Moreover, this biphasic stimulant and sedative effect of ethanol observed in zebrafish has been correlated with brain ethanol concentrations [33]. In our research, a biphasic response was also observed between the exposures carried out during the photophase and the darkness phase, despite the ethanol concentration used for all trials was the same (1% v/v). In fact, zebrafish response during the photophase and darkness phase corresponded with those reported before during exposure to high and low-moderate concentrations of ethanol, respectively. These different responses suggest that ethanol uptake from the water might have been different at different ZTs, affecting the final dose in blood and brain and therefore eliciting different neurobehavioural effects, although further research will be needed to confirm this hypothesis. Our findings agree with earlier research in mice, showing more severe effects of ethanol during the active phase of this nocturnal species (i.e. during the dark phase), although the ethanol concentration in the blood and brain of mice was lower at night when compared to levels observed during daytime, when ethanol toxicity was lower [16]. In addition, Sato et al. [17] observed that the hypnotic duration of GABAergic drugs (including ethanol) was also higher during the active phase of mice. Similarly, previous studies have also found daily variations in the behavioural response of zebrafish and gilthead seabream exposed to sublethal concentrations of anaesthetics. In these cases, the greater effects (shorter induction time of anaesthesia and longer recovery time) were also observed during the day, coinciding with the active phase of the animals [28,23], suggesting a link between the daily rhythms of activity and toxicity. Actually, in gilthead sea bream, day-night variations of MS-222 in plasma following exposure were reported, being higher during the day, when the induction of anaesthesia occurred more rapidly [34]. Acute stress responses in gilthead sea bream is also rhythmic, as physiological and oxidative stress indicators rose higher when the stressor was applied at night (resting phase), further fostering the concept of time-dependent mechanisms in the control of neurophysiological responses [35].

The daily differences in toxicity reported here could be the result of the existence of daily rhythmicity at different physiological levels, from the absorption, distribution and excretion mechanisms to the metabolising pathways [36]. In zebrafish liver the ethanol metabolising machinery is similar to the system described in mammals and involves the enzymes ADH and ALDH [13,14,37,38]. Tran et al. [10] found that acute exposure to ethanol differentially affected the activity of ADH and ALDH in zebrafish liver. Thus, low doses of ethanol (0.25–0.5% v/v) caused a dose-dependent increase in ADH activity whereas higher concentrations (1% v/v) provoked a decrease in the activity of this phase I detoxification enzyme, correlating with the biphasic response observed in the effect of ethanol on neurobehavioural response, thus suggesting an effect of ethanol on its metabolic pathway. However, the existence of circadian variations in the expression of genes encoding the enzymes involved in ethanol detoxification had not been investigated so far. Under an LD cycle, temporal variations were observed in this study, although these differences were not statistically significant. Likewise, *in vitro* metabolism of ethanol by hepatic ADH in mice did not show daily differences, despite the existence of toxicity rhythms *in vivo*, suggesting that time-dependent differences in the sensitivity of the nervous system to ethanol might be one of the factors involved in ethanol chronotoxicity [16]. In DD, however, we found circadian rhythmicity in the expression of all the studied genes (*adh5*, *adh8a*, *aldh2*.*1* and *aldh2*.*2*), with the acrophases located at the end of the subjective night, whereas the lowest levels of ethanol toxicity were found in the middle of the darkness phase, in both the lethality and behavioural tests. However, no studies were carried out at protein level in the present investigation and a direct cause-response effect between detoxification rhythms and ethanol toxicity cannot be concluded at this point. Similarly, in the malaria mosquito, Balmert et al. [39] also found rhythms in detoxification genes, as well as in total enzyme activity, but the acrophases of these rhythms were not coincident and gene expression rhythms did not always correlated to biochemical activity, supporting previous evidence suggesting the existence of temporal changes in post-transcriptional and post-translational processes that may affect the daily rhythm of protein abundance [40]. Therefore, rhythms in the detoxification mechanisms may contribute to the chronotoxicity of ethanol, although circadian variations at uptake, absorption, and/or pharmacokinetics level cannot be ruled out as they might also affect this rhythmicity. The existence of circadian rhythms in the absence of external cues indicated that the expression of genes involved in ethanol metabolism in zebrafish is clock-controlled, despite the fact that gene expression did not show significant rhythmicity in LD. Previous research in mammals has revealed that the activity levels of ocular ADH and ALDH differs between individuals kept under an LD or in DD [41] and that some members of the ALDH family are able to directly absorb UV-light, playing a protective role in the cornea against excessive production of reactive oxygen species (ROS) [42]. However, to our knowledge, there is a lack of information regarding the immediate effects of light in hepatic isoforms investigated in the present study. Similarly, the expression of several detoxification and lipid metabolism genes in zebrafish liver has been recently reported to be both light- and clock-controlled, with the acrophases located in the interphase between night and day under an LD cycle or at the end of the subjective night when fish were kept in constant darkness [43,44], coinciding with the acrophases observed in the present research. In fact, both detoxification genes and key transcription factors regulating their expression have been reported to show rhythmic expression in zebrafish [43]. The existence of such rhythms would confer an adaptive advantage to fish and previous studies have found that the expression of detoxification genes coincided with the onset of the animals’ active phase, when the risk of exposure to toxicants would be higher [45]. For instance, it is well recognized that food is an important source of naturally occurring toxins [46]. In addition, previous research has reported that hepatic ADH and ALDH are involved in retinol and retinal metabolism. Retinol (vitamin A) is converted into retinoic acid which binds a nuclear receptor signaling pathway that is involved in growth and development regulation and epithelial maintenance [47]. Furthermore, aldehyde dehydrogenases have a varied range of biological functions, including the regulation of the metabolism of the neurotransmitter GABA and the elimination of reactive aldehydes derived from lipid peroxidation [48]. Therefore, circadian regulation of these enzymes in zebrafish may play additional roles in different metabolic pathways.

In conclusion, this research showed that ethanol toxicity exhibits daily rhythmicity in zebrafish larvae and adults. In particular, ethanol effect was more detrimental when fish were exposed in the morning, whereas the toxicological and neurobehavioural responses were attenuated when exposure was carried out at night. In addition, our results revealed that the expression of genes involved in ethanol detoxification is under circadian regulation in zebrafish liver. Finally, zebrafish is a model species widely used in chronobiology and the present investigation contributes to increase our knowledge about time-dependent effects of this drug, a topic that had been little explored despite the potential impact on public health and social care research.

## Supporting information

S1 File Mortality in zebrafish larvae exposed to 4% ethanol. Mortality rate for zebrafish larvae exposed to 4% ethanol for 1 h at ZT2, 6, 10, 14 and 18. Data are shown as the mean ± SE (n = 60 larvae/ZT).
(XLSX)
